# Primary Paraganglioma of the Lung in an Asymptomatic Patient

**DOI:** 10.7759/cureus.25562

**Published:** 2022-06-01

**Authors:** Brandon C Warren, Tanner Bond, Richard Hessler, John Boldt

**Affiliations:** 1 Internal Medicine, University of Tennessee at Chattanooga, Chattanooga, USA; 2 Pathology, Erlanger Health System, Chattanooga, USA; 3 Pulmonary/Critical Care, Erlanger Health System, Chattanooga, USA

**Keywords:** suspicious lung mass, pheochromocytoma, neuroendocrine tumor, incidentaloma, paraganglioma

## Abstract

Arising from the autonomic paraganglia of the neuroendocrine system, paragangliomas are rare neoplasms that are derived from the embryonic neural crest. Primary paragangliomas of the lung are exceedingly rare, with little known about their origin. Here we present a 47-year-old female presenting in 2021, one year after a COVID-19 infection, with symptoms of tachycardia, shortness of breath, and palpitations which she associated with presumed long COVID-19. An imaging workup noted a 1.5 cm nodule in the lingula of the left lung. She then had surgical resection of the nodule, which was found to be a primary lung paraganglioma. A follow-up dotatate positron emission tomography (PET) CT noted no residual disease, and genetic testing was negative for known mutations. This case demonstrates the need for close monitoring with follow-up for incidental findings in order to provide a timely and accurate diagnosis in accordance with guideline criteria.

## Introduction

Paragangliomas are neuroendocrine tumors derived from the embryonic neural crest [1]. They may derive from the parasympathetic or sympathetic ganglia. The parasympathetic paragangliomas are generally found in the head and neck, most commonly arising from the carotid body. Sympathetic paragangliomas are more commonly found in the abdomen and rarely arise in the thorax, most commonly in the middle and posterior mediastinum. Parasympathetic paragangliomas are more likely to be non-functioning compared to sympathetic paragangliomas, which are more likely found to be functioning by secreting catecholamines [1].

Paragangliomas are rare tumors with an incidence rate combined with pheochromocytomas of 0.8 per 100,000 person-years [2]. Primary pulmonary paragangliomas (PPP), in comparison, are exceedingly rare, with 22 cases documented previously based on 2012 chart reviews [3]. PPP generally presents as an incidental finding on imaging, with the majority of tumors found to be non-functioning, with no catecholamine secretion [4]. This tumor may present as incidental findings on imaging studies of the lung, and follow-up imaging is warranted. Here we present a 47-year-old female who was seen in the pulmonology clinic for shortness of breath on exertion and tachycardia with a recent history of coronavirus disease 2019 (COVID-19) infection a year prior and a known 4 mm left lung upper lobe nodule on computerized tomography (CT) of the chest in 2014.

## Case presentation

In October of 2021, a 47-year-old female with a past medical history of follicular thyroid adenoma with surgical removal in the year 2000 and a 4 mm nodule in the lingula of the lung noted on chest CT in the year 2014 presented to the pulmonology clinic for evaluation of tachycardia, shortness of breath and dyspnea on exertion for the past four to six months. She had previously been evaluated with an electrocardiogram (ECG) in the emergency department and a stress test that showed no evidence of myocardial ischemia. She reported a previous history of COVID-19 in 2020, but since that period, she has had no symptoms of tachycardia or shortness of breath. Because of her new-onset dyspnea and tachycardia, she presented to the pulmonology clinic for evaluation. 

**Figure 1:**
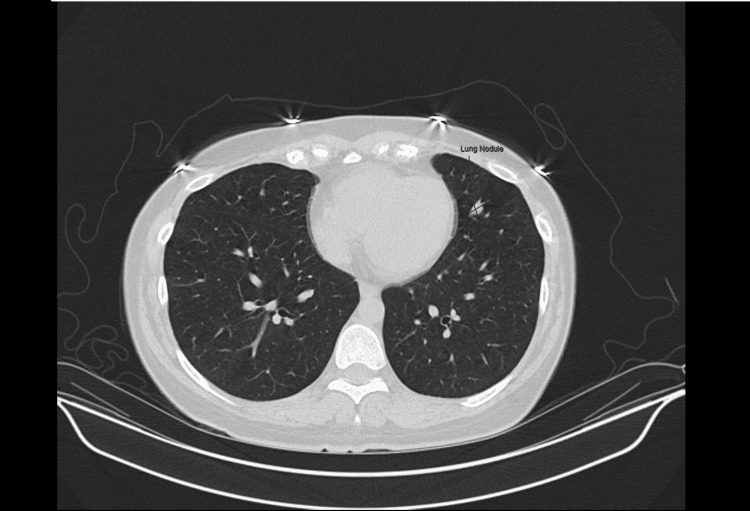
Computerized tomography of the chest in 2014 showing a 4 mm left upper lobe lingular lung nodule

**Figure 2:**
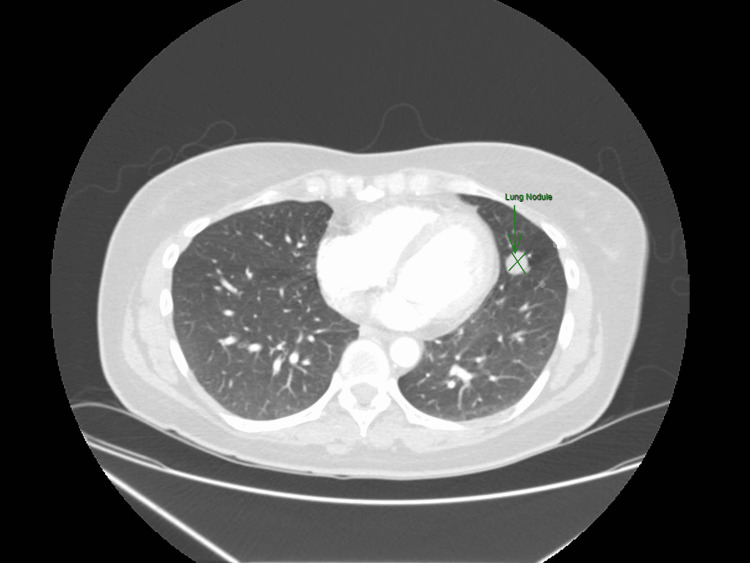
Computerized tomography of the chest in 2021 showing an increase in the lung nodule size to 15 mm

**Figure 3:**
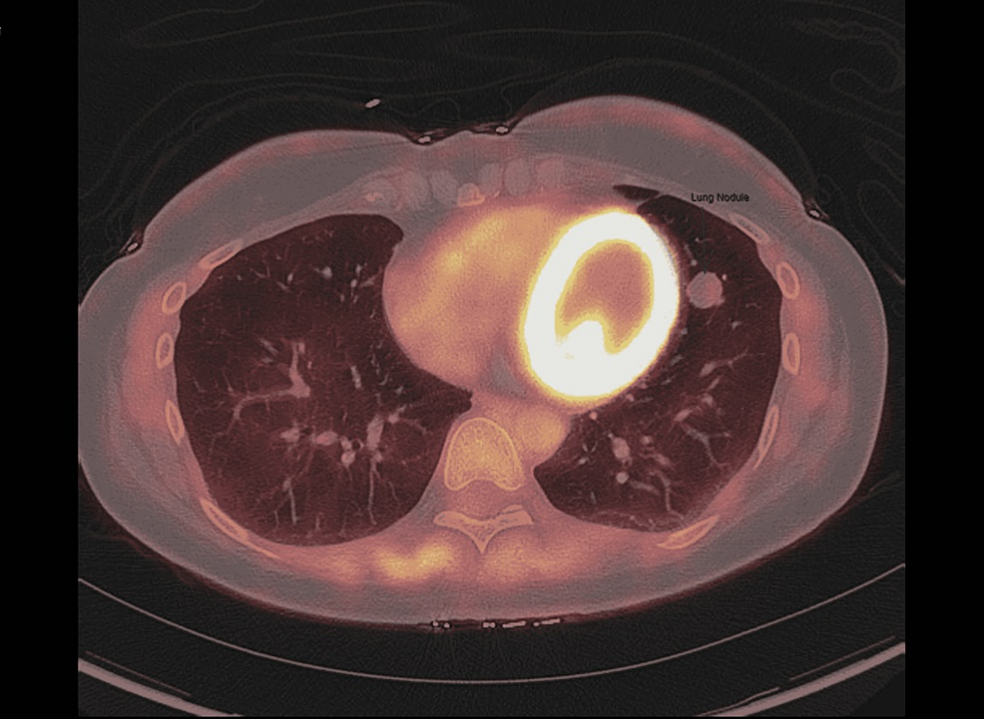
Positive emission tomography scan of the chest showing no uptake of I-123 in the left upper lobe lingular nodule

Initial work-up included a computed tomography pulmonary angiogram (CTPA) of the chest to rule out pulmonary embolism, which found no acute findings but did note an increase in the size of the known 4 mm noncalcified nodule in the lingula of the left upper lobe (Figure 1). This nodule was noted to have increased from 4 mm to 15 mm on repeat imaging at her clinic appointment for the work-up of her tachycardia and dyspnea (Figure 2). The patient's case was presented at the local thoracic tumor case conference. A single-photon emission computerized tomography (SPECT) scan was performed, which showed no focal uptake of radioactive iodine I-123 in the left lung or elsewhere in the body. The patient was referred to cardiothoracic surgery, which recommended the patient undergo a positron emission tomography (PET) CT that showed no significant metabolic activity within the lingular nodule of the left lung (Figure 3). 

**Figure 4:**
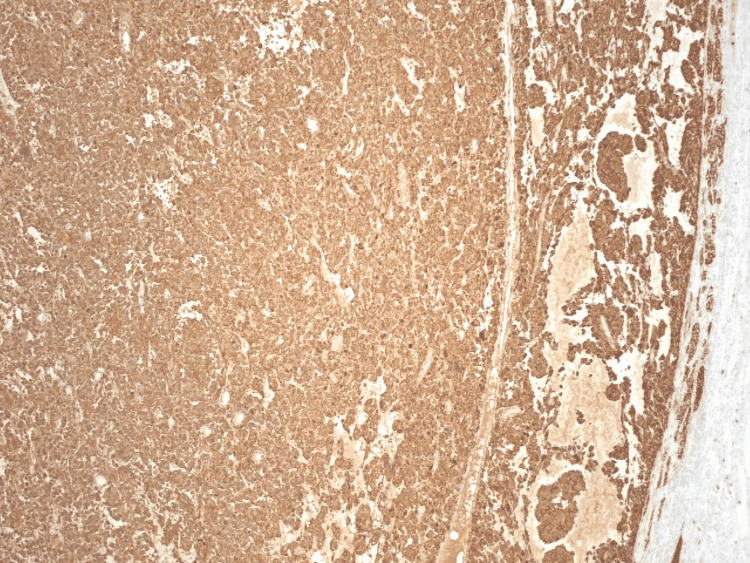
Immunohistochemistry stain showing positive chromogranin

**Figure 5:**
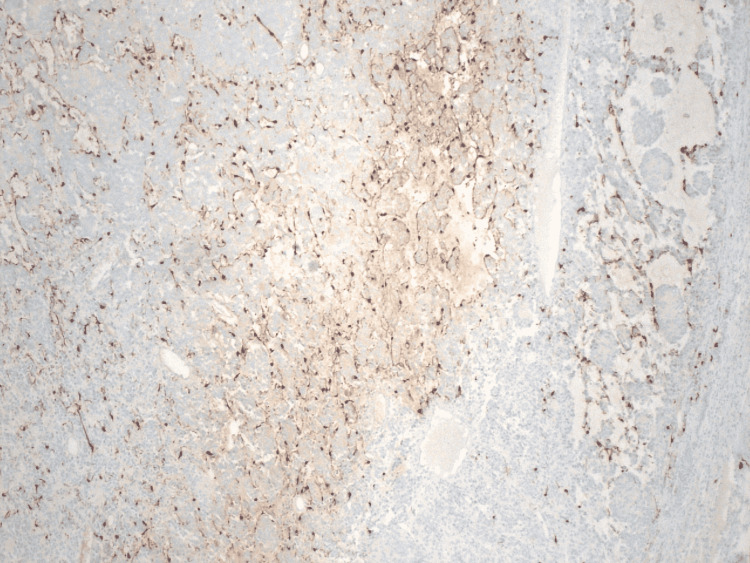
S-100 stain showing sustentacular cells

A discussion between pulmonology and cardiothoracic surgery determined that because of the increase in the size of the pulmonary nodule, it would be better to be resected at that time rather than wait for it to further increase in size. The patient underwent a video-assisted thoracoscopic surgery with a left upper lobe lingular wedge resection in December of 2021. The patient did well in the postoperative period with no immediate recurrence of shortness of breath or chest pain, or any symptoms of infection. The pathology report noted a 15 x 12 x 12 mm nodule with negative borders described as tan to gray-white with diffusely positive chromogranin and synaptophysin (Figure 4). It also noted positive S-100 in sustentacular cells and negative CK AE1/3 (Figure 5). Based on these findings, the pathology report stated that the nodule was found to be a paraganglioma.

The patient was then referred to hematology/oncology for evaluation, who ordered plasma-free metanephrines and plasma catecholamines, of which all were within normal limits. She was referred to medical genetics and underwent genetic testing with no genetic mutations noted, including multiple endocrine neoplasia type 2 (MEN2). A referral was placed to a regional academic medical center hematology/oncology department for further evaluation, and the recommendations were to organize follow-up surveillance with repeat CT of the chest at six-month intervals along with plasma free metanephrines. She also was referred to cardiology for further evaluation of her ongoing palpitations and intermittent tachycardia.

## Discussion

A review of the literature up to this point seems to indicate that a majority of known paragangliomas are sporadic, though it has a predilection to appear in middle-aged females [3]. Functional paragangliomas are primarily related to the sympathetic nervous system and are generally found within the paravertebral ganglia. These actively secrete catecholamines and present with symptoms similar to pheochromocytomas with symptoms including hypertension, headache, tachypnea, tachycardia, and anxiety [1]. Parasympathetic non-functional paragangliomas are typically discovered incidentally through a chest radiograph or CT scan. Since they do not secrete neuroactive hormones, if they were to present with symptoms, it would likely be related to the compression of nearby structures due to the mass effect.

PPP is still exceedingly rare in literature, and there are no pathognomonic radiology findings that would have led to consideration for this disease preoperatively. With our patient, there has been no indication of local invasion or metastasis, which is consistent with literature citing that only about 10% of paragangliomas are malignant [5]. Metastasis in PPP still has not been reported in the literature, so this would lead to the logical assumption that these can be managed with a surgery-only approach combined with surveillance imaging without chemotherapy or radiation.

Fleischner Society guidelines are the standard for the management of most incidentally found pulmonary nodules found on imaging [6]. In general solid nodules less than 6 mm do not need follow-up unless the patient is at high risk, such as smokers, in which case a 12-month follow-up chest CT is also within guidelines. When this patient's nodule was originally assessed in 2014, the nodule was only noted to be 4 mm, and as a result, there was no further follow-up needed at that time. Because of the increase in the size of the pulmonary nodule to 15 mm from 4 mm between 2014 and 2021, a discussion between pulmonology and cardiothoracic surgery was held, and it was elected to have the nodule surgically resected for curative intent rather than biopsy the nodule. Based on the Fleischner criteria, additional options may have included a repeat CT of the chest in three months. This demonstrates a need for discussion between providers in order to determine the best clinical outcome for a patient. In this case, it was elected to have the nodule resected as it had tripled in size over a seven-year period, and it was felt that the patient would have the best chance for a positive clinical outcome with a resection now rather than after further monitoring and the possible development of new medical comorbidities. Because a PPP was not in the initial consideration before resection, screening markers for a functioning PPP were not obtained.

## Conclusions

Primary pulmonary paragangliomas are exceedingly rare tumors of the neuroendocrine system. The majority of these are non-secreting and slow-growing, as presented in this case. Many are found incidentally on imaging studies and should be followed up based on Fleischner Society pulmonary nodule guidelines. In this case, the option to perform a wedge resection of the lingula to remove the nodule was elected based on the increase in the size of the nodule over a seven-year period. After resection of the nodule, genetic testing should be performed to rule out disorders, such as MEN2. This case demonstrates the need to have a broad clinical diagnosis in the management of incidental imaging findings and close follow-up in accordance with guideline criteria.
